# Does *Triatoma brasiliensis* occupy the same environmental niche space as *Triatoma melanica*?

**DOI:** 10.1186/s13071-015-0973-4

**Published:** 2015-07-10

**Authors:** Rita de Cássia Moreira de Souza, Gabriel H Campolina-Silva, Claudia Mendonça Bezerra, Liléia Diotaiuti, David E Gorla

**Affiliations:** Laboratório de Triatomíneos e Epidemiologia da Doença de Chagas, Centro de Pesquisas René Rachou, Fundação Oswaldo Cruz, CEP 30.190-002 Belo Horizonte, Minas Gerais Brazil; Secretaria de Saúde do Estado do Ceará,, Fortaleza, Ceará Brazil; IMBIV-CONICET, Casilla de Correo 495, 5000 Córdoba, Argentina

**Keywords:** Triatominae, Geometric Morphometrics, DNA barcode, Chagas disease, Geographic distribution, Ecological niche modelling

## Abstract

**Background:**

Triatomines (Hemiptera, Reduviidae) are vectors of *Trypanosoma cruzi*, the causative agent of Chagas disease, one of the most important vector-borne diseases in Latin America. This study compares the environmental niche spaces of *Triatoma brasiliensis* and *Triatoma melanica* using ecological niche modelling and reports findings on DNA barcoding and wing geometric morphometrics as tools for the identification of these species.

**Methods:**

We compared the geographic distribution of the species using generalized linear models fitted to elevation and current data on land surface temperature, vegetation cover and rainfall recorded by earth observation satellites for northeastern Brazil. Additionally, we evaluated nucleotide sequence data from the barcode region of the mitochondrial cytochrome c oxidase subunit I (CO1) and wing geometric morphometrics as taxonomic identification tools for *T. brasiliensis* and *T. melanica*.

**Results:**

The ecological niche models show that the environmental spaces currently occupied by *T. brasiliensis* and *T. melanica* are similar although not equivalent, and associated with the caatinga ecosystem. The CO1 sequence analyses based on pair wise genetic distance matrix calculated using Kimura 2-Parameter (K2P) evolutionary model, clearly separate the two species, supporting the barcoding gap. Wing size and shape analyses based on seven landmarks of 72 field specimens confirmed consistent differences between *T. brasiliensis* and *T. melanica*.

**Conclusion:**

Our results suggest that the separation of the two species should be attributed to a factor that does not include the current environmental conditions. However, as the caatinga is a biome that has existed in the area for at least the last 18,000 years, past conditions might have had an influence in the speciation process. The DNA Barcoding approach may be extended to these members of the subfamily Triatominae.

## Background

The epidemiological importance of the Triatominae, a subfamily of blood-sucking bugs, derives from its capacity to transmit *Trypanosoma cruzi*, the etiological agent of Chagas disease, one of the most important vector borne diseases affecting around 8 million people in Latin America [1]. At present, 148 triatomine species are recognized, whose taxonomic knowledge reflects a balance between the use of traditional approaches and a wide variety of evolutionary interpretations [2–8].

*Triatoma brasiliensis sensu lato* Neiva, 1911 is one of the most important species for Chagas disease transmission and the main vector of *T. cruzi* in semi-arid areas of Northeastern Brazil, colonizing both natural and artificial ecotopes [9, 10]. This species shows remarkable chromatic variation across its geographic distribution, and indeed, it was concluded that *T. brasiliensis* is a complex of species.

Multidisciplinary studies have indicated that the *Triatoma brasiliensis* complex is a monophyletic group [11, 12], comprising four species, one of which includes two distinct subspecies. The relevant taxa in the group are *T. brasiliensis brasiliensis* Neiva, 1911; *T. brasiliensis macromelasoma* Galvão, 1956; *Triatoma juazeirensis* Costa & Felix, 2007; *Triatoma melanica* Neiva & Lent, 1941 and *Triatoma sherlocki* Papa *et al.* 2002. Each member of the group can be identified by external morphological characteristics and a taxonomic key was recently published [12].

Correct taxonomic identification of these insects is important because triatomine bugs behave differently in terms of their adaptation to artificial environments. Some species are primarily found in sylvatic ecotopes, where they live associated with small nest-building mammals or birds. Others invade and can even colonize the peridomestic environment and/or the human dwellings, exposing domestic mammals and humans to the infection of *T. cruzi* [13–17]. Knowledge about the environmental space occupied by the triatomine species is an important factor for the development and/or improvement of vector control and surveillance strategies [18].

Currently, complementary methods to the classical taxonomy have been used in the comparative study of Triatominae species, such as geometric morphometric and molecular techniques. Wing shape is increasingly utilized in species identification and characterization [19] and in distinguishing sibling species [20–22].

Wings are excellent structures for studying morphological variation because the intersections of the wing veins provide many well-defined landmarks suitable for morphometrics, and the metric properties of the wing provide precise quantitative information for the identification of species complexes [23, 24] and within-species variations [25]. In triatomines, the study of morphological variation by geometric morphometrics has been helpful to solve taxonomic problems [20, 26, 27], to analyze the house reinfestation phenomenon [28, 29] and to distinguish cryptic species [23, 30, 31].

Variations that are not expressed phenotypically ascribes importance to molecular studies for the comprehension of systematic issues. In this sense, the DNA sequencing has been widely used for identification, determination of phylogenetic relationships and inferences. The DNA Barcoding method is a system of universal taxonomic identification, fast with free access, based on the variation of a part of the sequence of the mitochondrial gene cytochrome c oxidase subunit I (CO1) [32]. This method remains in constant evaluation and, among other groups, the use of the CO1 sequences has shown promise for species-level identification in insects, including cryptic species of triatomines [33–36] and other insects [37–41].

After the confirmation of the species composition of the *T. brasiliensis* complex, we started exploration about the causes of the speciation process within the complex. Guided by the general question on speciation causes, this study aims at verifying the hypothesis that *T. brasiliensis* and *T. melanica* occupy different environmental niche spaces as a consequence of the speciation process within the *T. brasiliensis* complex. For the first time, we report findings on DNA barcoding as a tool for the identification of these species, and to complement, we also report the analysis of wing geometric morphometrics.

## Methods

### Insects

The study was based on a dataset that combined localities where sylvatic specimens of *T. brasiliensis* and *T. melanica* were collected in the municipalities of Ceará and Minas Gerais, respectively, by our study group, and localities listed in recent bibliographic records (Table 1) [42–50]. Specimens were collected among rocky crevices by manual capture performed by ourselves, assisted by the personnel of the Chagas Disease Control Programme (PCDCh). Species identification was based on diagnostic characters [12] and supported by geometric morphometric and DNA barcoding.

**Table 1:** *Triatoma brasiliensis* and *Triatoma melanica* collection sites

Espécie	Localities (Estate)	Coordinates	
	Cachoeira do Júlio (CE)	−5.7797386568	−40.2799790369
*T. brasiliensis*		−5.7943959655	−40.2933588601
		−5.7957817887	−40.2964052389
		−5.7981479146	−40.29790065
		−5.7810796276	−40.2868991416
		−5.7756609478	−40.2914290151
		−5.7745058503	−40.290306677
		−5.7707807942	−40.2898106334
		−5.7663582906	−40.2777547391
	Mutuca (CE)	−5.7639571507	−40.3154218119
		−5.7639571507	−40.3154218119
		−5.7510605317	−40.3074732164
		−5.7529589079	−40.3078567957
		−5.7546603659	−40.3073459943
		−5.7538574416	−40.3104142327
		−5.7571074248	−40.3129680423
		−5.7545489571	−40.3086008597
		−5.7634536703	−40.3141293975
		−5.760313526	−40.3188447104
		−5.7593630921	−40.3152848227
		−5.7509785672	−40.3037708967
		−5.7505637589	−40.3032372062
		−5.7540372706	−40.302983254
		−5.7556812626	−40.3038719055
		−5.7594241925	−40.3044493114
	Caico (RN) [42, 50]	−6.4580555556	−37.0552
		−6.4586111111	−37.1027777778
		−6.45	−37.0833333333
	João Costa (PI) [42]	−8.5625	−42.2615
	Jaguaruana (CE) [42–44]	−4.8338888889	−37.4652
		−4.7908333333	−37.8211111111
		−4.7911111111	−37.8416666667
		−4.8358333333	−37.78
	Independencia (CE) [46]	−5.3833333333	−40.19
	Tauá (CE)	−6.0030555556	−40.2927777778
	Monteiro (PB) [49]	−7.8913888889	−37.1169444444
	Mãe d’Água (PB) [49]	−7.2527777778	−37.4327777778
	Lagoa Grande (PE) [49]	−8.9852777778	−40.3055555556
	Serra Talhada (PE) [49]	−7.9858333333	−38.2938888889
	São José (PB) [49]	−6.8666666667	−38.6333333333
	Santa Cruz (PB) [49]	−6.5258333333	−38.0566666667
	São Francisco (PB) [49]	−6.6147222222	−38.0894444444
	Jaguaruana (CE) [45]	−4.8666666667	−37.8666666667
	Morada Nova (CE) [47]	−5.1066666667	−38.3725
	Caicó (RN) [48]	−6.4583333333	−37.0977777778
	Santa Fé (CE)	−5.79861	−41.39784,
		−5.76942	−41.38401
		−5.80709	−41.38547
		−5.807068529	−41.3656128355
		−5.8066754454	−41.3636071708
		−5.8066401856	−41.3632278149
	Betânia (CE)	−5.7996606198	−41.3164524623
	Merejo do Angico (CE)	−5.7970432243	−41.3675663039
		−5.7972957301	−41.3678649052
		−5.7897151958	−41.3760729586
	São Bento do Incra (CE)	−5.8217903855	−41.3052064709
		−5.8216638327	−41.305179086
		−5.82219407	−41.3027239521
		−5.5448297123	−41.2859612517
		−5.8244361104	−41.3031084099
		−5.8244361104	−41.3031084099
	Umburana (CE)	−5.8067181193	−41.2836429863
		−5.8065007531	−41.2837779525
		−5.8038787237	−41.2834378894
		−5.8041314457	−41.2836642239
		−5.8040407996	−41.2837543239
		−5.8011474622	−41.2754126471
		−5.8010926881	−41.2756382863
		−5.8009294863	−41.2758185293
		−5.8009476357	−41.2757914787
	Morada Nova do Pedro (CE)	−5.8183702425	−41.2940818102
		−5.8185060277	−41.2940279364
		−5.818089856	−41.2940901997
		−5.818090084	−41.2939908626
		−5.8171389269	−41.290710548
		−5.8166869508	−41.2906101799
		−5.8150583777	−41.290886418
		−5.8170585434	−41.2942142713
		−5.8169040397	−41.2984763947
		−5.8169040397	−41.2984763947
		−5.8167956776	−41.2984039009
		−5.8169766832	−41.2983501321
		−5.8152342328	−41.2930812692
		−5.8148007181	−41.2928183904
		−5.8149275626	−40.2927193428
	São Cristóvão (CE)	−5.844311984	−40.3427462733
		−5.8428131129	−40.3492810785
		−5.8429752726	−40.3495433628
		−5.8429754463	−40.3494711161
		−5.8429395965	−40.3493355668
		−5.8419807752	−40.3494055181
		−5.8400112339	−40.3485609342
	Várzea do Touro (CE)	−5.8144558964	−40.3549657346
	Jasmin do Aluísio (CE)	−5.7988865422	−40.3379969007
		−5.7992664716	−40.337961675
		−5.7997549705	−40.3379086446
		−5.7997185812	−40.3379988614
		−5.8005141668	−40.3381181298
	Canadá (CE)	−5.8209865096	−40.3164207266
		−5.825308579	−40.3168371717
		−5.826086614	−40.3167306155
	Açude Novo do Satiro (CE)	−5.7763231261	−40.3489331747
		−5.7814635364	−40.3321586694
		−5.7817173389	−40.3319154535
		−5.7814635364	−40.3321586694
		−5.7831583815	−40.3344652846
		−5.7814548089	−40.3358699676
		−5.7844424461	−40.3383782891
		−5.7845597824	−40.338477895
		−5.7843793519	−40.3382878407
		−5.7765014709	−40.3499991176
	Benfica do Incra (CE)	−5.810092187	−40.3109319444
		−5.8098751729	−40.3109133814
	Mutuquinha (CE)	−5.7803063443	−40.3007856841
		−5.7803967433	−40.3008039507
		−5.7777991803	−40.3055749036
		−5.7776364086	−40.3055655008
		−5.7832888298	−40.3095426674
		−5.7851697965	−40.3096282653
		−5.7833611194	−40.3095699239
		−5.7808520905	−40.3112256831
		−5.7809642108	−40.3135827822
		−5.7817054004	−40.3137650925
		−5.7801335005	−40.313002945
		−5.7800790903	−40.3130660297
		−5.7797858352	−40.3147268772
		−5.7780757056	−40.3111651136
		−5.7777506427	−40.3109386159
		−5.7770410578	−40.3127339502
		−5.7761366159	−40.3127499273
		−5.7753138628	−40.3087206715
		−5.7755129561	−40.3086669487
		−5.7753138628	−40.3087206715
		−5.7752779365	−40.3086122294
	Morada Nova do Tomaz (CE)	−5.8240075662	−40.2849374675
		−5.8243344098	−40.2843873297
		−5.8247497376	−40.2846953218
		−5.828842364	−40.2905566441
		−5.8287520269	−40.2905112833
		−5.8335974273	−40.2914886694
		−5.8328556691	−40.2915501889
		−5.8256738966	−40.2918769394
		−5.8250568901	−40.2927515183
		−5.8254811314	−40.2931137214
		−5.8254539578	−40.2931317208
		−5.8273886598	−40.2934522275
		−5.8260122409	−40.2941986356
	Cachoeira dos Pedrosas (CE)	−5.7753947305	−40.2811883403
		−5.7776457504	−40.2776084476
	Belo Horizonte do Alfredo (CE)	−5.7507140311	−40.3243757442
		−5.7501845917	−40.3264783952
		−5.7514881386	−40.3337140541
		−5.7510529737	−40.334164515
		−5.7553014006	−40.3351225211
		−5.7554069432	−40.3363959345
		−5.7548096631	−40.3365570712
		−5.7536164371	−40.3363104873
		−5.7532550612	−40.3361471122
		−5.7645775066	−40.3440835719
		−5.7468382666	−40.3108406169
*T. melanica*	Espinosa (MG)	−14.9430862989	−42.8228295697
		−14.9429787099	−42.7587081764
		−14.9425322811	−42.7583317976
		−14.9429146471	−42.7586252043
		−14.9432840482	−42.7585191465
		−14.9428410758	−42.7575758586
		−14.9261111111	−42.8191666667
	Monte Azul (MG)	−15.0649784693	−42.9291096633
		−15.0651120404	−42.9288944895
		−15.0651088061	−42.9285411508
		−15.0650568078	−42.9287834333
		−15.063781528	−42.9306277034
	Porteirinha (MG) [42]	−15.7430555556	−43.2830555556
	Urandi (BA) [36, 50]	−15.2777777778	−43.0941666667
		−14.5	−42.6833333333

### Geometric morphometrics

**Fig. 1:**
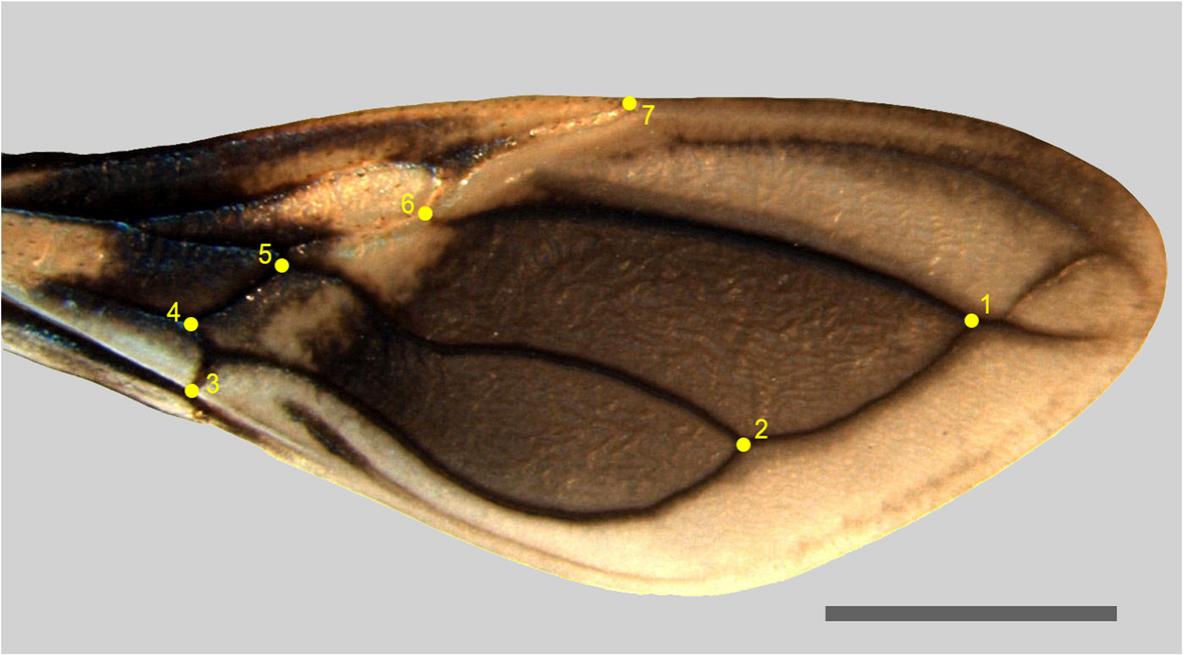
Right wing of *Triatoma melanica* with the seven type I landmarks used in analysis. The same configuration was taken for *T. brasiliensis*. Scale bar = 300 mm

Right hemelytra (forewings) from 36 insects of each species (18 females and 18 males), from the municipalities of Tauá (CE) and Espinosa (MG), were mounted between microscope slides. The wing images were captured using a digital camera (Carl Zeiss®Axiocam MRC 3.0) coupled to a Zeiss® Stemi SV6 stereomicroscope. Seven type I landmarks (venation intersection, according to Bookstein, 1991) were digitized, as shown in Fig. 1. In order to reduce the measurement error, landmark coordinates were recorded three times on each wing and the average of the results was calculated.

Centroid size (CS), an isometric estimator derived from coordinate data, was computed as a measure of overall wing size. It is defined as the square root of the sum of the squared distances between the centre of the configuration of landmarks and each individual landmark [51]. The centroid size values of different species and sexes were compared by multiple linear regression using the “lm function” of R. The presence of atypical points (outliers) was verified and a 95 % confidence interval for the estimated regression coefficients was constructed. In this analysis, males and females were examined separately to check for the presence of sexual dimorphism.

Generalized Procrustes Analysis (GPA) superimposition algorithm [52] was used to produce shape variables. Here, both non-uniform (‘Partial Warps’) and the uniform component were used as shape variables, which describe respectively the local and global variation as deviations from a consensus shape [53]. In order to explore the wing shape variation among species, a multivariate discriminant analysis was performed on shape variables and the main results were shown by a factorial map of the first two discriminant factors. Mahalanobis distances were also computed from the same variables and used to illustrate morphological divergence among groups (species and sexes) by a dendrogram constructed according to the UPGMA algorithm. Statistical significance of these distances was assessed by non-parametric analyses based on permutation tests (1000 runs), and corrected by the Bonferroni method for P ≤ 0.05.

The residual relationship between shape and size variables was evaluated using a multivariate regression analysis of the first two shape discriminant factors against the centroid size values. This procedure allowed measurement of the contribution of size to shape variation (allometry), where statistical significance was assessed according to the non-parametric method based on permutation (1000 runs) [54].

We used the modules COO, TET, MOG, COV and PAD from the CLIC package (freely available at http://mome-clic.com/) to collect landmarks, to compute centroid size and shape variables, as well as to evaluate the residual relationship between shape and size variables, and to generate discriminant models and Mahalanobis distances. For comparing overall wing size among groups and to elaborate quantile-plots of CS values, the lm function of R was used. The UPGMA dendrogram based on Mahalanobis distances was constructed using the neighbor module from the PHYLIP package (by Joe Felsenstein, available at http://evolution.genetics.washington.edu/phylip.html).

### Molecular analysis

Genomic DNA was extracted from individual specimen legs (stored at −70 °C) using the Wizard Genomic DNA Purification System (Promega, Madison, WI), re-suspended in 30 μl of DNA Rehydration Solution (10 mM Tris pH 8.0, 1 mM EDTA pH 8.0) and then stored at −80 °C until amplification by standard polymerase chain reaction (PCR). A fragment of 658 bp in the 5′ end of the CO1 gene was amplified using the primers LCO 1490 (5′-GGTCAACAAATCATAAAGATATTGG-3′) and HCO 2198 (5′-TAAACTTCAGGGTGACCAAAAAATCA-3′) [55], following the thermal cycling conditions: an initial denaturation step at 94 °C for 3 min, 30 cycles at 94 °C for 1 min, 45 °C for 1 min and 72 °C for 1 min, with a final extension at 72 °C for 10 min. The 20 μl PCR reaction mixes included 10 μl of ultrapure water, 2 μl of 10x PCR buffer, 0,8 μl of each primer (10 μM), 2 μl of dNTP (2,5 mM), 2 μl of MgCl2 (50 mM), 0,4 μl of Taq polymerase (5 U/μl) and 2 μl of the DNA template (10 ng/μl). The PCR products were visualized on a 2 % agarose gel and selected for direct sequencing, where both DNA strands were sequenced using the BigDye Terminator v3.1 Cycle Sequencing Kit (Applied Biosystems, Inc., Foster City, California, USA), following the manufacturer’s protocol on an ABI 3730XL Automated DNA Analyzer (Applied Biosystems) available at the Oswaldo Cruz Institute, FIOCRUZ (Rio de Janeiro, Brazil).

All sequences were aligned with the Clustal W and edited using the Geneious R6 software (Biomatters Ltd, Auckland, New Zealand®). The consensus sequences analysed in this study are deposited on GenBank under the accession numbers KJ580486 to KJ580495. Afterwards, we used MEGA v. 5.2 [56] to carry the sequence analyses. Intraspecific and interspecific genetic distances (sequence divergence) were calculated using the Kimura 2-Parameter (K2P) nucleotide substitution model [57]. In this approach, we also quantified the number of variables sites among sequences, as well as the nucleotide frequencies. A neighbor-joining (NJ) tree was constructed based on the K2P distances for representation of relationships among the analysed sequences, and the reliability of each node was estimated using the bootstrap method with 2,000 replicates. The tree was rooted using sequences of *Triatoma sordida* Stål, 1851 from Minas Gerais, Brazil (G. H. Campolina-Silva, unpublished data) as an outgroup.

All barcoded specimens have been deposited in the Coleção de Vetores da Doença de Chagas (COLVEC), Centro de Pesquisas René Rachou/FIOCRUZ.

### Ecological niche modelling

Ecological niche modelling methods were used for the study of the geographic distribution of biological species in general and triatomines in particular [17, 58, 59], using environmental variables as proxies of species niche dimensions. Two main approaches were used as environmental data sources: the Worldclim database [60] and remotely sensed variables recorded by earth observation satellites.

Previously published studies on geographic distribution of the *T. brasiliensis* species complex [10, 12, 17] used occurrence coordinates of specimens mainly collected during the 1980s and 1990s, before the species identification within the *T. brasiliensis* complex. To avoid confusion of occurrence points of possibly misclassified specimens, the present study of the geographic distribution of *T. brasiliensis* and *T. melanica* was based on an estimation of the environmental space from occurrence points where each species was collected either by our study group or taken from recent bibliographic records (15 for *T. melanica* and 140 for *T. brasiliensis*). The number of occurrence points in this study is smaller than the set analysed in previous studies (for example Costa *et al.* 2002) [61], as those studies used several occurrence points that in our consideration were initially identified as *T. brasiliensis*, but afterwards were synonymized to other specific names within the group complex. We used a set of occurrence points that have no possibility of taxonomic misidentification as *T. brasiliensis brasiliensis*. We consider that this conservative selection of occurrence points should show the distribution area of the species without errors. No absence points were used, but rather a random selection of 1000 background points. No biotic or abiotic barriers are known or can be imagined for the dispersion of the studied species, so that the accessible area for them is the whole study area. Although flying and/or walking are the most frequent mechanisms for active (and short ranged) dispersion in triatomines, passive dispersion (especially through human migrations) is the main mechanism for long range dispersion, as a number of very early studies demonstrated and recently confirmed using genetic markers [62].

A great variety of methods exist to estimate species distribution models. The maximum entropy is one of the most frequently used, because of the simplicity of use of the Maxent software. However, based on the simplicity criteria [63], the possibility of testing explicit assumptions, and on the equivalence between the results obtained using Maxent and Poisson point process models [64], we used a generalized linear model with a binomial link through the glm function of the R package.

A major objective of studies examining niche overlap is to determine if two species occupy the same niche. Even if two or more niches are identical, there will be some differences in the data purely by chance. To rule out detecting two niches as different, that actually only differ due to sampling variation, niche comparisons between two species must be done statistically to determine whether the same probability distribution describes the niche of two species, or whether there is evidence of some difference [65]. The estimated distribution models for the *T. melani*ca and *T. brasiliensis* were compared using the Schoener index of niche overlap D, an index varying between 0–1 (no niche overlap and identical niche, respectively), and used to perform niche equivalency and similarity tests [66]. We followed the framework proposed by Broennimann *et al.* (2012) [67] (based on Warren *et al.* 2008) [68], and specifically an ordination method (PCA-env) to estimate niche overlap, as the method disentangles the dependence of species occurrence from different climatic conditions (correcting for relative availability of environments) and from environmental data resolution. For the niche equivalency test all occurrences were pooled and randomly split into two datasets, maintaining the number of occurrences as in the original datasets, and the niche overlap statistic D was calculated. The process was repeated 100 times and a histogram of simulated values was constructed. If the observed value of D falls within the density of 95 % of the simulated values, the null hypothesis of niche equivalency cannot be rejected. For the niche similarity test we randomly shift the entire observed density of occurrences in one range and calculate the overlap of the simulated niche with the observed niche in the other range. The test of niche similarity was also based on 100 repetitions. If the observed overlap is greater than 95 % of the simulated values, the entity occupies environments in both of its ranges that are more similar to each other than expected by chance. All calculations were carried out using an R script produced by Broennimann *et al.* (2012) [67], and appropriately modified.

Environmental variables included in this study were day and night land surface temperature (LST) and vegetation index (NDVI) estimated from raster images produced by the MODIS sensor during the period 2009 – 2013 (downloaded as granules h12v09, h13v09, h13v10, h13v11, h14v09, h14v10, h14v11 from the http://e4ftl01.cr.usgs.gov/MOLT/ site); rainfall estimated by the Tropical Rainfall Measuring Mission (TRMM) during the period 2005 – 2013 (downloaded from the Goddard Earth Science site http://mirador.gsfc.nasa.gov/collections/TRMM_3B43__007.shtml) and elevation (produced by the Shuttle RT Mission and downloaded as a digital elevation model (DEM) from the CGIAR Consortium for Spatial Information at http://www.cgiar-csi.org/data/srtm-90m-digital-elevation-database-v4-1). Temporal resolution was 8, 16 and 30 days for LST, NDVI and rainfall, respectively. The spatial resolution at the data sources were 1 km for LST, 250 metres for NDVI, 0.5° (nominally 25 kms at the Equator) for rainfall and 90 metres for the DEM. All imagery was reprojected to a common geographic projection with WGS84 datum and resampled to 250 metres spatial resolution. As some points of triatomine collection were closer than 250 m apart, the number of occurrence points considered for *T. melanica* decreased to 8 and 132 for *T. brasiliensis*. A total of 200 images of LST (day and night) dates, 100 of NDVI dates and 108 monthly rainfall time series were processed calculating average and standard deviation of the time series for the period 2009–2013. The ecological niche model was thus based on 9 environmental predictors. A separate model for each species was calculated using the glm function of R version 3.0.2. Variables were selected to minimize the AIC (Akaike Information Criteria) using the stepAIC package. Colinearity among predictor variables was evaluated using the variance inflation factor (using vif function of the R car package) and a variable remained in the model if vif <10. Area under the receiver operation curve was calculated for model evaluation estimating partial AUC using the pROC package of R [69] that take into account the criticisms raised by Lobo *et al.* 2007 on the use of AUC [70]. Cross-validation (through the cv.glm function) was used to measure the robustness of model estimation, using K = 100 for *T. brasiliensis* and K = 8 for *T. melanica*.

## Results

### Morphometric analysis

**Fig. 2:**
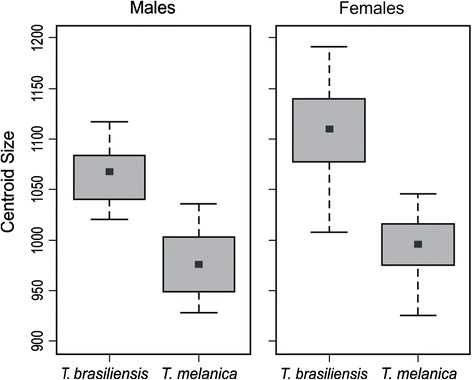
Wing centroid size variation among males and females of *T. brasiliensis* and *T. melanica*. This shows the sexual size dimorphism. The group mean is shown as a box between the quartiles (25th and 75th percentiles) and the standard deviations are shown as lines. Units are pixels

As expected, there was a significant difference in centroid size values in both sexes and species (P < 0.001) (Fig. 2). A consistent sexual size dimorphism was observed, where the female wings were larger than the males. Wing size was largest in *T. brasiliensis*, as observed by Costa and colleagues [42].

**Fig. 3:**
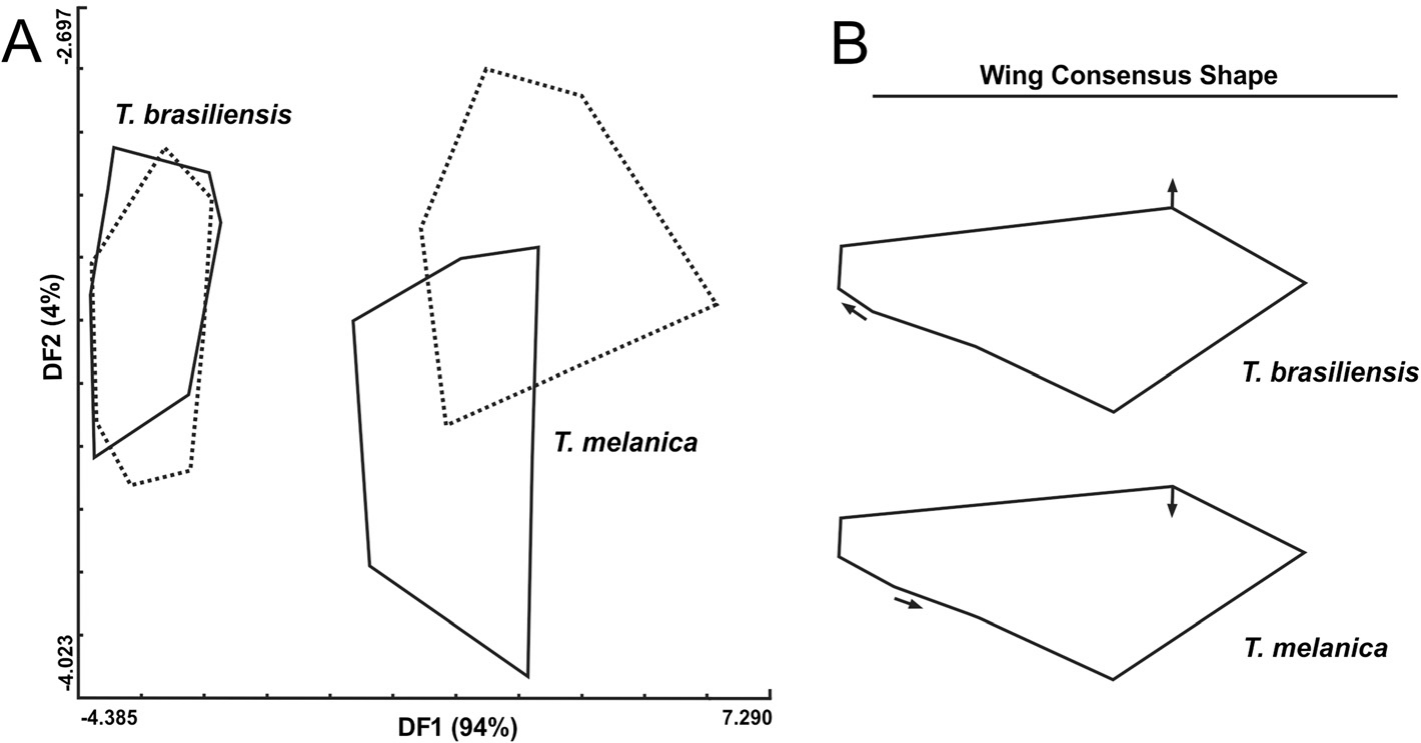
Shape variation in *T. brasiliensis* and *T. melanica*. (A) Factorial map showing the distribution of specimens in the plane of the two discriminant factors derived from discriminant analysis of the shape variables. Polygons correspond to each group under study, where continuous polygons enclose males and dotted polygons enclose females. DF1 and DF2 are the first and second discriminate factors, and their corresponding percentage contribution to the total shape variation is shown in parentheses. (B) Wing consensus shape obtained after the Generalized Procrustes Analyses (GPA). Arrows indicate the major differences in wing shape of *T. brasiliensis* and *T. melanica*

**Fig. 4:**
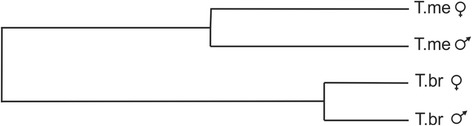
UPGMA dendogram based on Mahalanobis distances derived from shape analyses. Observe a clear separation between *T. melanica* (T.me) and *T. brasiliensis* (T.br)

The first two discriminant factors (DF1 and DF2) explained 94 % and 4 % of wing shape variation, respectively. *T. brasiliensis* and *T. melanica* were significantly different (P < 0.01) and well separated in a factorial map of discriminant analysis along the DF1 axis (Fig. 3a). *T. melanica* wings were narrow when compared with *T. brasiliensis*, and the landmarks 3 and 7 better contributed to the conformational changes in wing venation (Fig. 3b), and are important in the discrimination of these species. Significant Mahalanobis distances could be found between species and sexes after the permutation test corrected by Bonferroni (P <0.05), except for males and females of *T. brasiliensis*. The UPGMA dendrogram derived from these distances showed a clear separation between the species (Fig. 4). The multivariate regression analyses of the first two shape discriminant factors (DF1 and DF2) against the centroid size values revealed no significant contribution of size to shape variation (no allometric content, p = 0.19).

### DNA Barcoding analysis

A total of 10 CO1 sequences were generated. There were no detected insertions, deletions or presence of stop codons. The DNA barcode region analysed in this study comprised 564 bp, where the nucleotide composition between *T. brasiliensis* and *T. melanica* showed themselves to be similar, with an overall average of nitrogenous bases being 30.4 % for Thymine (T), 28.1 % for adenine (A), 24.4 % for Cytosine (C), and 17.1 % for Guanine (G). A total of 56 variation sites was observed (mainly at the third codon position) between the sequences, 55 (98.2 %) of these were partially informative and in 43 (76.8 %) there were fixed nucleotide substitutions, i.e. specific sites that are common to all sequences where there was exact replacement of a certain nucleotide between the two species. Transitions were more frequent than transversions (Table 2), with the transition C ↔ T being the most commonly observed.

**Table 2:** Summary of the variation along of the CO1 sequences analyzed

	Nucleotides	Variables sites	TS	TV
1st	188	13	9	0
2nd	188	14	10	3
3rd	188	29	18	3
Total	564	56	37	6

Variables sites, transition and transversion rates among *Triatoma brasiliensis* and *Triatoma melanica*. TS: number of transitions; TV: number of transversions

In order to evaluate the interspecific genetic divergence, the pairwise intra- and interspecific genetic distance (K2P) was calculated. The average intraspecific K2P distance was 0.006, ranging from 0 to 0.009 in *T. brasiliensis* (0.005 ± SD0.002) and from 0.002 to 0.013 in *T. melanica* (0.008 ± SD 0.004), whereas the average K2P distance between these two closely related species was fifteen times higher (0.099 ± SD 0.003, ranging from 0.094 to 0.103), supporting the barcoding gap, as proposed by Hebert and colleagues [71]. The values of the K2P distances are shown in Table 3.

**Table 3:** Pairwise intra and interspecific genetic distances, based on the Kimura 2-Parameter model

SAMPLES	1	2	3	4	5	6	7	8	9	10
1. T.me (KJ580495)	**–**									
2. T.me (KJ580494)	0,002	**–**								
3. T.me (KJ580493)	0,011	0,013	**–**							
4. T.me (KJ580492)	0,009	0,011	0,002	**–**						
5. T.br (KJ580490)	0,101	0,101	0,099	0,099	**–**					
6. T.br (KJ580486)	0,103	0,103	0,101	0,101	0,002	**–**				
7. T.br (KJ580488)	0,103	0,103	0,101	0,101	0,005	0,004	**–**			
8. T.br (KJ580491)	0,101	0,101	0,099	0,099	0	0,002	0,005	**–**		
9. T.br (KJ580487)	0,097	0,096	0,094	0,094	0,007	0,005	0,009	0,007	**–**	
10. T.br (KJ580489)	0,097	0,096	0,094	0,094	0,007	0,005	0,009	0,007	0	**–**

T.me: *Triatoma melanica*; T.br: *Triatoma brasiliensis.* The numbers inside the parentheses correspond to the *GenBank accession numbers*

**Fig. 5:**
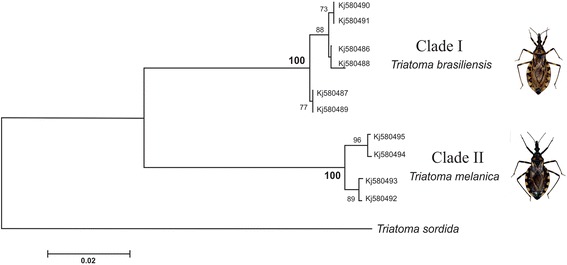
Neighbor-joining tree based on K2P distances of CO1 sequences from *T. brasiliensis* and *T. melanica*. The NJ tree was rooted using sequences of *Triatoma sordida* and the bootstrap value of 2,000 replicates is shown on each node. Scale bar = 0.02

A neighbor-joining (NJ) tree constructed based on K2P distances (Fig. 5) shows two distinct clades strongly supported by a bootstrap value of 100 %, comprising a group formed by all *T. brasiliensi*s individuals and another by *T. melanica*. Hence, our results indicate that these species might be recognized by the barcode region evaluated in this study.

### Geographic distributions

The model estimating the distribution of *T. brasiliensis* (n = 132) showed the average of LSTd and accumulated rainfall, and the variability of LSTd and LSTn and rainfall were all highly significant (P < 0.001) in describing the species occurrence sites (Table 4). The occurrence chance of this species is positively related with LSTd and variability of LSTn, and negatively related with accumulated rainfall and variability of LSTd. Model fit is very good, with high partial AUC (pAUC) = 95 % (CI95 of 91.3 – 97.2) and low cross validation error (0.041).

**Table 4:** Generalised linear model fit

*Triatoma brasiliensis*	*Triatoma melanica*		
Coefficient	Estimate	Coefficient	Estimate
Intercept	−1.96e + 02***	Intercept	−6.527e + 02***
AVGLSTD	1.038e-02***	AVGLSTD	4.126e-02***
SDAVGLSTD	−4.507e-04***	AVGNDVI	2.916e-03**
SDAVGLSTN	8.526e-04***	SDAVGRAIN	−2.154e-02*
AVGRAIN	−3.291e-03***	DEM	1.430e-02***
AUC	0.976		0.989
Cross Validation	0.0409		0.00854

Geographic distribution of *T. brasiliensis* (132 occurrence points), *T. melanica* (8 occurrence points) and 1000 (independent for each model) background points. The remotely sensed environmental variables are AVGLSTD (average of diurnal land surface temperature), AVGRAIN (average of annual accumulated rainfall), AVGNDVI (NDVI average), DEM (elevation), SDAVGLSTD and SDAVGLSTN (standard deviation of diurnal and nocturnal land surface temperature, respectively), SDAVGRAIN (standard deviation of annual accumulated rainfall). *P < 0.05; **P < 0.01, ***P < 0.001

The model for *T. melanica* (n = 8) showed that elevation, annual averages of LSTd, NDVI and variability of accumulated rainfall were the significant variables (Table 4). The occurrence chance of this species is positively related with elevation and averages of LSTd and NDVI, and negatively related with the variability of accumulated rainfall. Model fit was high, with pAUC = 98 % (CI95 of 97.2 – 99.4) and low cross validation error = 0.0085.

**Fig. 6:**
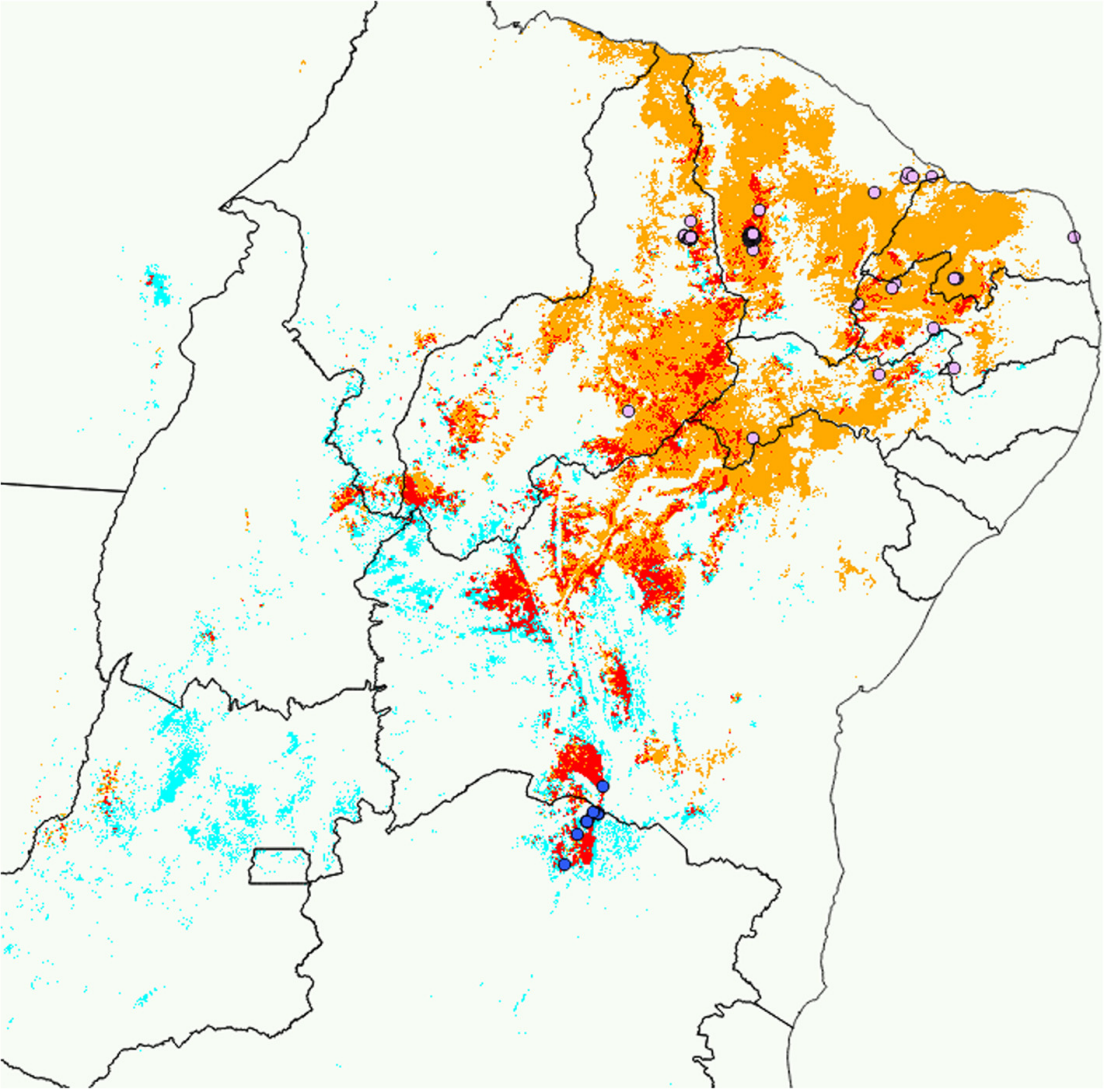
Modeling Disease Vector Occurrence. Geographic distribution of *T. brasiliensis* (orange), *T. melanica* (light blue) estimated by the generalized linear model for each species, and area where both models overlap (red). *T. brasiliensis* occurrence points (pink circles) and *T. melanica* occurrence points (blue circles)

The model that predicts the potential distribution of *T. brasiliensis* identifies the main caatinga region as an area with similar environmental properties as the one where this specie was collected. The model for *T. melanica* predicts a sparse area towards the west (Goiás and Tocantins) and the northeast into the main caatinga region. Although map prediction identified different areas for the species, there are wide overlapping areas of model occurrence prediction. Figure 6 shows the prediction for the *T. brasiliensis* and *T. melanica* models, together with the area where the two models overlap.

Niche comparison using principal component analysis showed that the two first principal components explain 58 % of the variation included in the 9 environmental variables used for the study. Niche overlap was estimated as D = 0.291. This value is larger than expected by chance, thus leading to acceptance of niche similarity (P > 0.05), although leading to rejection of niche equivalency (P < 0.05).

## Discussion

Based on similarities in morphology, geographical distribution profile, epidemiological significance and phylogenetic relationships, a review [2] analyzing the systematics and evolution of the Triatominae subfamily, list the probable existence of 8 species complexes within the genus *Triatoma* Laporte, 1832. Among these, the *T. brasiliensis* complex has been continuously evaluated and is currently composed by *T. melanica, T. juazeirensis, T. sherlocki* and *T. brasiliensis*, the latter having two subspecies, *T. brasiliensis brasiliensis* and *T. brasiliensis macromelasoma* [12].

Among the five members of this complex, the subspecies *T. b. brasiliensis* has been considered as the main concern in terms of Chagas disease transmission, because this subspecies is the most widespread and shows the highest rate of domestic capture and of natural infection by *T. cruzi* [9, 10]. On the other hand, to date, *T. melanica* has been found almost exclusively in wild environments, and collected specimens have been shown to be restricted to municipalities in the northern region of Minas Gerais and the southern region of Bahia [12].

Analysis involving Triatominae mtDNA sequences are regularly used in order to elucidate phylogenetic relationships among closely related Triatominae species. In this respect, the most widely used markers include fragments of gene encoding proteins such as cytochrome b [11, 34, 36, 72, 73] and subunits of ribosomal RNA (12S and 16S) [33, 74–76]. The present study shows the effectiveness of DNA barcoding in identifying species of the *T. brasiliensis* complex and reinforces its contribution to classic taxonomy. The analyzed CO1 fragment was sufficient to discriminate *T. brasiliensis* and *T. melanica*, showing that these species are separated by a large genetic distance. The discrimination of these species can be well represented by the NJ tree with bootstrap values of 100 % for the two identified clades. This shows that our analysis of pair wise distance-based K2P within the *T. brasiliensis* complex supported a clear barcoding gap between the intra- and interspecific variation [76].

In a recent study, Justi *et al.* conclude that DNA barcoding is not applicable for the identification of Southern American *Triatoma* species, which may have diverged recently, because they observed at least one intraspecific genetic distance greater than interspecific distance in assessed species [77]. The authors mention that to be considered appropriate to identify species, intraspecific distances must always be lower than interspecific ones. Unlike the findings of Justi *et al.*, the results of this study show that the CO1 barcode region is a robust marker for differentiating this species complex, and it may constitute a valuable tool for both epidemiologic studies and for Chagas disease control, including subspecies differentiation, as observed by Vendrami *et al.* [49]. Furthermore, this methodology can be especially useful for identifying immature stages, whose traditional taxonomic method is made impossible in smaller nymphs, especially considering the species complexes that exhibit wide morphological and chromatic variation.

The barcode sequences result found is congruent with the geometric morphometric analysis. Centroid size variation was as significant in the interspecific analysis as it was in the separation between males and females. Size variation related to gender, with females of both species larger than males, was expected with the presence of sexual dimorphism being a known characteristic in triatomines [78]. The morphometric analyses of wing was able to discriminate the two members of the complex (DF1 = 94 %), allowing assignment without error of each single wing to its corresponding taxon, and producing a pattern of relationships in agreement within known genetic distances between populations, as observed by Costa and colleagues [42]. As a result of there being no allometric trends for wing shape comparisons between *T. brasiliensis* and *T. melanica*, it is suggested that the change in this structure’s shape should mainly reflect the variation of genetic origin [69]. Furthermore, the recorded sexual dimorphism suggests stability and adaptation of both species to the sylvatic ecotope, without evidence of exposure to environmental stress conditions, which may lead to reduction in size of females, as proposed by Dujardin *et al.* 1999 [79].

The ecological niche model using elevation and current data on land surface temperature, vegetation cover and rainfall showed that elevation, the average of LSTd, accumulated rainfall and variability of LSTd and LSTn are the environmental variables that best describe the geographic distribution of *T. brasiliensis*, whereas elevation, the average of LST, NDVI and variability of accumulated rainfall are the ones best describing the distribution of *T. melanica*. The result for *T. melanica* is probably less reliable, because of the small sample size of occurrence points, and restricted to a small area. Conversely, the prediction map for *T. brasiliensis* is very reliable, and indicates a potential distribution particularly associated with the caatinga biome. Of particular importance for the objective of this study is the niche comparison between the species. Results show that niches are similar but not equivalent, suggesting that *T. brasiliensis* and *T. melanica* share similar environmental constraints but that a different set of variables within the environmental niche space restrict their distribution. It is interesting that the model for *T. brasiliensis* predicts occurrence where *T. melanica* was collected, and the model of *T. melanica* predicts occurrence (although loosely) where *T. brasiliensis* was collected. Both species are associated with the caatinga region and have rupicolous habits. No difference between the species at the microscale is expected within the macro landscape of the caatinga, but this is an aspect that should be verified under field conditions. In evolutionary terms, the results of this study suggest that the separation in two different species should be attributed to a factor that does not include the current environmental conditions. However, as the caatinga is a biome that has existed in the area for at least the last 18,000 years [80, 81], past conditions might have had an influence in the speciation process.

The potential geographic distribution maps obtained differed with previously published studies [17, 82]. The differences are attributed to a) our use of a different set of occurrence points of specimens, based on criteria of minimum chance of species misidentification, a weakness that old records, previous to the species identification within the *T. brasiliensis* complex might possess, b) our use of current environmental conditions of temperature, vegetation and rainfall (measured by sensors onboard earth observation satellites, reflecting the land cover situation within the last 5 years of an area that shows an important landscape modification within the last 20 years), instead of the Bioclim database [60] that reflect past conditions (averaged between 1950–2000), through an interpolation process of ground meteorological stations, that in the area were extremely sparse during the considered period and might produce low reliability for the temperature and rainfall variables for the area, and c) our approach used for the whole study area as potentially accessible by the species, without imposing political boundary limits to map predictions.

Despite the successful vector control programme of *Triatoma infestans* in most parts of the southern cone countries of South America, native vectors persistently reinfest insecticide-treated households, and sylvatic triatomines maintain disease transmission without colonizing human dwellings. In these scenarios, fine-scale vector studies are essential to define epidemiological risk patterns and clarify the involvement of the little-known triatomine taxa in disease transmission. These eco-epidemiological investigations, as well as the planning and monitoring of control interventions, rely on accurate taxonomic judgments. The problems of cryptic speciation and phenotypic plasticity illustrate this need.

## Conclusion

Although DNA barcoding is a straightforward approach, it is applicable for identifying species of the *T. brasiliensis* complex and contributes to classic taxonomy. The analyses of wing shape was able to differentiate the two members of the complex, and showed that the species are not under stressful conditions. As a result of there being no allometric trends in the wing shape comparison between *T. brasiliensis* and *T. melanica*, it is suggested that the change in this structure’s shape should mainly reflect the variation of genetic origin. In evolutionary terms, the result of the environmental niche space occupied by the studied species suggests that their separation as two different entities should be attributed to a factor that does not include the current environmental conditions. However, as the caatinga is a biome that has existed in the area for at least the last 18,000 years, past conditions might have had an influence in the speciation process.
